# Influence of Sleep Characteristic Changes on Nurses’ Quality of Life during Their Transition to Practice: A Prospective Cohort Study

**DOI:** 10.3390/ijerph19010573

**Published:** 2022-01-05

**Authors:** Kyoungja Kim, Youngjin Lee

**Affiliations:** 1 Department of Nursing, College of Medicine, Inha University, Incheon 22212, Korea; asteria43@inha.ac.kr; 2 College of Nursing, Ajou University, Suwon 16499, Korea

**Keywords:** sleep hygiene, quality of life, nurse

## Abstract

Aim: To explore the effect of changes in sleep characteristics on changes in quality of life during the transition period of new graduate nurses. Background: Sleep problems among nurses are associated with negative physical and psychological consequences. Methods: This prospective cohort study was conducted at a tertiary hospital in South Korea. Participants included 88 newly graduated nurses. Data were collected twice, prior to shift work and after 4 months of working as a nurse, via online structured self-report questionnaires created using Survey Monkey from March 2018 to February 2020. A generalized linear mixed model was used to analyze the influence of changes in sleep characteristics on quality of life. Results: A generalized linear mixed model showed that changes in the subjective quality of sleep, subjective health perception, and daytime dysfunction influenced quality of life changes during the transition. This implies that deterioration already existed. From their undergraduate period to four months after they began working as nurses, a significant decrease was observed in the quality of sleep. Participants’ quality of life significantly decreased. Conclusions: Changes in the quality of life of new graduate nurses may show deterioration with a significant drop in subjective sleep quality. Institutions should improve existing work adaptation programs provided during new graduate nurses’ transition to practice by including information on changes in nurses’ health caused by changes in sleep characteristics and sleep quality.

## 1. Introduction

Globally, the turnover rate of registered nurses is high [1] and varies across countries, such as New Zealand (44.3%) [2]; Canada (19.9%) [3]; and Australia (15.1%) [4]. In the US, the turnover rate is up to 26.8% for registered nurses [5], 17–18.1% for new graduate nurses within the first year [6,7], and 26% within the first two years [8]. In South Korea, according to the Hospital Nurses Association of Korea [9], in 2018, the turnover rate of new graduate nurses within a year of appointment was as high as 43.5% at tertiary general hospitals. This is markedly higher than the turnover rate of all nurses (11.6%) in South Korea [9]. Such a high turnover rate among new graduate nurses substantially increases the burden on hospitals and society, and the associated costs are as high as USD 82,000–88,000 [10]. It also increases the workload of remaining nurses, and the continuous changes it causes in the skill mix reduce the quality of nursing and affect patient outcomes [11].

One of the main reasons for turnover among new graduate nurses is the lack of an appropriate transition process [9]. According to Opoku et al. [12], “the transition to practice for new graduate nurses was described as gradual and complex, involving a complete transformation particularly in the first year after graduation” (p. 2). During the transition process, new graduate nurses should achieve substantial adjustments from graduates to independent professional nurses [13]. Therefore, appropriate transition experiences are critical for new graduate nurses to retain their nursing jobs, particularly in the first 1–2 years [12]. However, Higgins et al. [14] stated that new graduate nurses struggle with successful transitions, such as lack of confidence in working independently, an inability to multitask, and establishing supportive interpersonal relationships. Consequently, during the transition process, new graduate nurses experience physical and psychological stress owing to the many changes they face in the hospital working environment [15,16]. One of the major changes is shift work, which is a typical nursing work characteristic [17]. Notably, over 80% of nurses work night shifts [9], and shift work induces changes in the lives of new graduate nurses. In terms of industrial safety, shift work is a major occupational hazard [18], and for nurses, it results in various health-related consequences by interfering with normal biological rhythms, including insufficient sleep, poor sleep quality, and fatigue, as well as a high risk of developing chronic diseases, such as hypertension and cancer [19,20,21,22,23]. Shift work maladaptation syndrome [24], which has been found in approximately 20% of shift workers, is characterized by sleep deprivation and fatigue and, above a certain level, results in an inability to retain the job. A previous study regarding the turnover of new graduate nurses showed that the burden of night shifts and the changeable lifestyle due to shift hours were the main causes of turnover, as they induced regret about nursing as a career choice [25].

Shift work in nursing has various consequences for both the physical and psychological health of nurses with the most common effect being a change in sleep quality. Notably, sleep disorders in shift workers occur more frequently than in non-shift workers [26]. As the nursing field involves shift work, sleep disorders in nurses have been found to cause work stress [27]; depression and anxiety [28]; general and mental health deterioration [29]; reduced work quality [30,31,32]; and turnover [33].

Sleep disorders in nurses involve sleep disturbances, irregular sleep characteristics, and abnormally increased sleep time. Such abnormal changes in sleep characteristics reduce sleep quality and eventually cause sleep deprivation [34]. Sleep disturbances, including obtaining too much or too little sleep, create a physical burden and increase the risk of all-cause mortality [35].

Therefore, examining changes in sleep characteristics among new graduate nurses is essential for improving the wellness and quality of life of nurses. Quality of life (QOL) is defined as a human’s impression of their situation in life in the context of the culture and value systems regarding their aims, expectations, standards, and concerns [36].

Moreover, the transition to practice is a critical period, during which new graduate nurses undergo many sudden changes as they face diverse work requirements [37], while also experiencing changes in sleep characteristics [38]. During this transition, nurses experience high levels of tension and responsibility, in addition to conflicts with colleagues and coworkers [37]. It is therefore necessary to identify changes that exert the most statistically significant influences on the quality of life of nurses during the early transition period.

While the transition period of new graduate nurses has not been clearly defined, the most widely accepted estimate is the first year of work; during the first three to six months of work, nurses are provided with various types of education and support systems by numerous institutions to promote their adaptation [39]. In South Korea, the first three months are generally regarded as the transition period, during which necessary education and support systems are provided to new graduate nurses [40]. In the fourth month of work, nurses begin to work independently of institutional support and education as they perform their duties and roles. Therefore, for the purposes of this study, the fourth month in the transition to practice for new graduate nurses was determined to be the period during which nurses’ quality of life changed the most.

This study aimed to examine changes in sleep characteristics and quality of life during the transition period of new graduate nurses and to determine the influence of such changes on their quality of life. Therefore, changes in sleep patterns and quality of life in the period between graduation and the four-month mark of working as a new graduate nurse were analyzed, in addition to identifying influencing factors of changes in the quality of life of new graduate nurses. In summary, the research questions are as follows: First, what are the differences between graduation and the four months after working on quality of life and sleep characteristics of new graduate nurses? Second, during the research period, did the change in sleep factors influence the change in quality of life for new graduate nurses?

## 2. Methods

### 2.1. Study Design

This prospective observational study assessed changes in the sleep characteristics of new graduate nurses as they started shift work during the “transition period” compared to the “graduate period” immediately after graduation and analyzed the consequent changes in their quality of life. The study procedure was reported in accordance with the Strengthening the Reporting of Observational Studies in Epidemiology (STROBE) guidelines.

### 2.2. Setting and Participants

The participants in this study were new graduate nurses who underwent on-the-job training at a tertiary hospital in South Korea. In order to control for other exogenous and endogenous confounding variables such as organizational characteristics, differences in transition training programs for new graduate nurses, and differences between hospitals, the cohort group included only new graduate nurses who worked in the hospital and were selected by the research team using convenience sampling. The selected hospital was a tertiary and university hospital with over 1100 beds in a metropolitan city in South Korea, and the bed-to-nurse ratio ranged from 2.0 to 2.5. This hospital operates a three-month on-the-job training and support program for new graduate nurses and a preceptorship program.

The target population comprised new graduate nurses who had not started clinical work as nurses and had not experienced shift work. The population accessed for this study comprised new graduate nurses participating in on-the-job training at a tertiary hospital before starting shift work. The participants voluntarily agreed to participate in the survey. All participants were nurses who performed 8-h shifts that sequentially rotated day, evening, and night. The required number of subjects was 62, as calculated using the G-power program 3.1.6, for *F*-tests (repeated measures, effect size = 0.15, α = 0.05, power = 0.95) by conducting two repeated tests. Assuming a 45% turnover rate up to follow-up monitoring four months later, a minimum sample size of 90 was required [9].

### 2.3. Data Collection and Procedure

Data were collected from the first and second surveys (after graduation and four months into shift work) via online self-report questionnaires created using Survey Monkey from March 2018 to February 2020. The first data collection was conducted eight times in total for graduating nurses at the hospital in March, April, June, and November in 2018 and March, June, August, and November in 2019. The second data collection was performed four months after the start of shift work from July 2018 to February 2020. A text message including a link to the online survey was sent to all participants who agreed to participate in this study after signing written informed consent forms. Each participant completed the online questionnaire independently and received a gift card worth USD 5. Participant information was coded electronically for data storage, and the investigator’s computer was password protected to prevent unauthorized access.

This study was approved by the Institutional Review Board of the researchers’ affiliated institution (IRB No. AJIRB-MED-SUR-17-411) and was conducted in accordance with the Code of Ethics of the World Medical Association (Declaration of Helsinki). To protect the rights of the participants, a joint investigator, unrelated to the participants, provided information about the study and collected informed consent forms from students who had voluntarily agreed to participate.

## 3. Measures

### 3.1. General Characteristics and Subjective Health Perception

General characteristics such as age, gender, religion, hobbies, location of upbringing, location of university, and grade point average (GPA) were measured by using a self-report questionnaire. Subjective health perception was measured using a single question about subjective health status, scored from very healthy (1 point) to very unhealthy (5 points).

### 3.2. Sleep Quality

Sleep characteristics were assessed using the Korean version of the Pittsburgh Sleep Quality Index (PSQI-K), translated and validated by Sohn et al. [41], and based on the original PSQI by Buysse et al. [42]. This questionnaire consists of 18 self-report items and measures seven domains: subjective sleep quality (one item, ranging from very good = 0 points to very poor = 3 points); sleep latency (one item, time (in minutes) to fall asleep); sleep duration (one item, actual sleep time); habitual sleep efficiency (three items, actual sleep time/(actual sleep time + sleep latency time) × 100); sleep disturbances (nine items, ranging from 0 = never to 3 = more than three times a week); use of sleep medication (one item, ranging from 0 = never to 3 = more than three times a week); and daytime dysfunction (two items, ranging from 0 = never to 3 = more than three times a week). In this study, the Cronbach’s alpha coefficients were 0.84 in the original PSQI-K [41] and 0.67 in this study.

### 3.3. Quality of Life

The participants’ quality of life was measured using the Korean version of the World Health Organization Quality of Life-Briefly (WHOQOL-BREF) [36,43]. WHOQOL-BREF consists of 26 questions: two on the overall quality of life and general health; seven on physical health; six on psychological health; three on social relationships; and eight on the work environment. The items were scored on a five-point scale, where 1 = Not at all, 2 = A little, 3 = Moderately, 4 = Very much, and 5 = Extremely. In the questionnaire, Questions 3, 4, and 26 were negative items, for which their results were subsequently inverted. A higher score indicates a higher quality of life. The reliability (Cronbach’s α) of the measurement tool was reported as 0.87 by Kim and Lee [44], while it was 0.92 in this study.

## 4. Data Analysis

Participants’ general characteristics, quality of sleep, and quality of life were analyzed using descriptive statistics. Continuous measures were summarized as means and standard deviations, while categorical variables were analyzed using frequencies and percentages.

In order to answer the first research question, we analyzed mean differences in the participants’ sleep characteristics and quality of life during the research period using paired *t*-tests. As exogenous variables, we measured several general characteristics, and among them, the subjective health status change between research periods was analyzed using a paired *t*-test.

For the second research question, we analyzed changes in sleep characteristics that had the greatest influence on changes in quality of life during the study period. In order to analyze this, we used a generalized linear mixed model. Generalized linear mixed models are generalized linear models that include one or more random effects, such as repeated measures within subjects [45]. For all analyses, the level of significance was set at *p* < 0.05.

## 5. Results

Of the 270 survey invitations sent, 109 (40.4%) questionnaires were answered in both rounds. The study excluded 17 questionnaires based on the exclusion criteria of working in a department that did not work in shifts and experiencing rotation or turnover as well as four questionnaires that contained responses to fewer than two scales; thus, 88 (80.7%) questionnaires were included in the final analysis.

### 5.1. Participants’ General Characteristics

The average age of the participants was 23.25, and the majority were female (71, 80.7%). Most participants did not have a religion (53, 60.2%), while 43 nurses (49.4%) replied that they had a hobby, with the most frequent type being exercise (20, 11.9%). The work departments were, sequentially, the medical (39, 44.4%), the surgical unit (37, 42.0%), and the emergency room (12, 13.6%; Table 1).

### 5.2. Participants’ Subjective Health Perception, Sleep Characteristics, and Sleep Quality

The mean score of subjective health perception in the graduate period was 1.96 ± 0.55; however, the score increased to 2.29 ± 0.62 during the transition period, representing a statistically significant difference (95% CI: (−0.48, −0.17), *p* < 0.001; Table 2).

The mean score of self-rated sleep quality in the graduate period was 1.26 ± 0.60, which increased to 1.66 ± 0.70 in the transition period, also a statistically significant difference (95% CI: (−0.31, −0.13), *p* < 0.001). In addition, the mean score of sleep disturbance in the graduate period was 4.96 ± 0.30 on a scale of 0–27 (with a higher score indicating a higher level of sleep disturbance), which increased to 7.07 ± 4.44 in the transition period, with a statistically significant difference (95% CI: (−3.14, −1.08), *p* < 0.001). Likewise, the mean score of daytime dysfunction was 1.12 ± 1.46 in the graduate period but increased to 1.63 ± 1.50 in the transition period, showing a statistically significant difference (95% CI: (−0.82, −0.21), *p* = 0.001); the measurement range was 0–6, with a higher score indicating a higher level of daytime dysfunction.

### 5.3. Participants’ Quality of Life

The mean score of quality of life, when measured within the range of 1–5, was 3.75 ± 0.43 in the undergraduate period but decreased to 3.18 ± 0.53 in the transition period, which was a statistically significant difference (95% CI: (0.46,0.68), *p* < 0.001; Table 3).

### 5.4. Factors Influencing Participants’ Quality of Life

The factors showing a statistically significant association with changes in quality of life were subjective health perception (95% CI: (−0.32, −0.03), *p* = 0.018), self-rated sleep quality (95% CI: (−0.39, −0.10), *p* = 0.001), and daytime dysfunction (95%CI: (−0.13, −0.01), *p* = 0.017; Table 4).

## 6. Discussion

This was a longitudinal cohort study for monitoring changes in sleep characteristics of new graduate nurses in the transition to practice and for determining consequent influences on their quality of life. The quality of sleep of new graduate nurses as measured at the fourth month after starting work during the transition to practice was 6.61 on average. Overall, a majority of nurses showed poor sleep quality, as the percentage of nurses with PSQI > 5, which indicates poor sleep quality according to the criteria of Buysse et al. [42], was 64.9%. The percentage was also higher than in the results of previous studies of sleep disorders among shift work nurses: 52.1% for experienced nurses in Spain [31], 33% for female nurses in Germany [46], 57% for shift work female nurses in Taiwan [47], and similar to the 65.1% for nurses in Brazil [48]. This result implies poor sleep quality for newly graduated nurses during the transition to practice. On the other hand, the percentage was lower than the 79.8% reported for nurses at small to medium hospitals in South Korea [32], although it should be noted that the work environment and age of the nurses were considerably different from those in this study.

The sleep quality of new graduate nurses was found to significantly decrease in the transition to practice. PSQI scores showed a statistically significant deterioration from 6.33 (±2.58) to 7.47 (±3.34). Notably, statistically significant differences were found in subjective sleep quality, sleep disturbances, and daytime dysfunction. Changing sleep characteristics are a natural phenomenon throughout an individual’s life. Previous studies of sleep characteristics according to changes in the life cycle and environment, via longitudinal monitoring, reported that sleep time decreased in females with aging from 0.9 min [49] to up to 0.11 h [50]. In comparison, the mean sleep time in this study at around four months showed a substantial fall from 6.39 (±1.76) h to 6.05 (±1.68) h. Such a sudden change in sleep characteristics is known to increase the rate of morbidity in various diseases, including cardiovascular diseases and metabolic syndromes [51], while increasing the mortality of underlying diseases [51]. Sleep change is also known to cause physical changes, such as cognitive impairment [52]. Another study of nurses showed that sleep disorders were correlated with depression, anxiety, and stress symptoms [53]. Even in the absence of morbidity of specific diseases or health deterioration, sleep disorders have been shown to negatively affect both physical and psychological health; subjective health perception is lowered so that individuals with sleep time ≤6 h or ≥10 h rate their health as poor to a greater degree than individuals with an optimal sleep time of 7–8 h [27,54]. Moreover, sleep disorders in nurses have been reported to decrease efficiency and productivity in nursing performance [55]. Nurses with sleep problems have a higher risk of medical error [56] and occupational injury [57]. These negative consequences of sleep disorders are presumed to limit adaptation proficiency of new graduate nurses during the transition process.

Previous studies have shown that not only physical factors, such as gender, fatigue, and physical activities, but also environmental factors, such as marital status and occupation, have statistically significant influences on changes in sleep characteristics [49]. Therefore, it is necessary to explore the factors related to sudden changes in sleep characteristics, as reported in this study. Regarding sleep quality in nurses, previous studies have consistently identified excessive work-related stress [27] and burnout [31] as the main factors of sleep quality deterioration in nurses. In addition, sleep quality in nurses showed a statistically significant decrease due to shift work itself [18], which is related to factors in the work environment such as nursing staffing, workload, work hours, and type of shift [32]. Notably, Lim et al. [58] reported that, among work environment factors, sleep disturbance in nurses was most markedly affected by interpersonal relationships. Cunningham et al. [59] reported that severe psychological distress induced changes in sleep characteristics such that affected individuals had a shorter or excessively longer duration of sleep than individuals with normal sleep characteristics. The transition to practice is a period that imposes drastic changes from new graduate nurses’ previous lifestyle to a three-shift routine; consequently, nurses simultaneously experience high levels of stress, an excessive burden, and difficulties in interpersonal relationships [60]. As these characteristics in the transition to practice act as risk factors for a sudden deterioration in sleep quality, new graduate nurses are likely to show sudden changes in sleep characteristics and sleep disorders. Therefore, interventions to prevent sleep disorders should be included in the transition program in order to support the adaptation of new graduate nurses.

The results of this study show that changes in the quality of life of new graduate nurses were negatively influenced by deterioration in their sleep quality. Although there are programs to support new graduate nurses and specialist nurses in the transition to practice, they mainly focus on confidence, competency, and knowledge of the work [15,61]. Despite the wide application of such programs, the high rate of resignations among new nurses is suggestive of the need for increased awareness among institutions. Notably, a study of nurses’ resignation experiences, based on in-depth interviews with new graduate nurses, reported that the key factor in the decision to resign was the physical burden of having to adapt to a different work and lifestyle, in addition to confidence, competency, and knowledge of the work itself [25]. Above all, nurse managers should be aware that healthy sleep is a critical factor and should encourage and support working nurses in achieving healthy sleep [55].

At the individual level, it is important that new graduate nurses be aware of the correlation between healthy sleep management and a successful transition to practice. Regarding the health management of nurses, Ross et al. [62] pointed out that, despite having in-depth health knowledge, nurses often fail to put that knowledge into practice, which necessitates not only education but also institutional strategies. Therefore, for effective health management, education on sleep hygiene for healthy sleep should be included as part of the program for their transition to practice. The necessary contents of sleep hygiene education programs are proper nutrition and time management, basic sleep management, knowledge of sleep disorder symptoms, responses to fatigue, and physical and psychological preparedness for shift work [55]. In cases in which new graduate nurses find it difficult to participate in such periodic educational sessions, personal consultations may be considered.

Providing a safe and healthy work environment is indeed an act of health promotion according to Cho and Han [63], who pointed out that excessive work was a factor that adversely affected the health of nurses and highlighted the need to create a healthy work environment and adjust the workload. As sleep problems may be due to overtime work, occupational stress, and rotating shift work [64], nurse managers should monitor the adaptation of new graduate nurses for an excessive level of sleep disorders. The institution should monitor new graduate nurses’ work hours to prevent overtime, and efforts should be made to maintain a healthy workplace.

## 7. Limitations and Suggestions for Future Research

This study was conducted with a prospective cohort design to examine sleep characteristics and the sudden deterioration in sleep quality in new graduate nurses transitioning to practice and to determine the most statistically significant factors that have negative effects on nurses’ quality of life. The findings provide basic data for developing programs for the successful transition of graduate nurses to clinical nursing, which constitutes the academic and practical significance of this study.

The limitations of this study are as follows: First, sleep characteristics and sleep quality measured in this study may not reflect actual sleep time, as they were based on a self-report questionnaire with potential subjective errors. Second, the external validity of this study is rather low because, to measure the quality of life, sleep characteristics, and quality of new graduate nurses in the transition to practice, convenience sampling was used, which limited the target population to new graduate nurses at a specific hospital in order to control exogenous variables (training of new graduate nurses, training period, nurse-to-patient ratio, hospital characteristics, etc.). In addition, factors having no statistically significant association with changes in quality of life were general characteristics (hobbies, religion, geographical area, and grade point average) and work-related characteristics (work department). Further studies are required to determine the effects of personal-and work-related traits. Finally, the results of this study are limited to a specific point in the nurses’ experience; that is, the surveys were completed before shift work and then in the fourth month after their work began. In the future, through the establishment of a prospective longitudinal cohort, it will be necessary to conduct a follow-up study on the effects of changes in sleep characteristics on personal and professional quality of life.

Future studies should, thus, apply a design that can more accurately measure sleep characteristics and sleep quality of nurses. Moreover, by using various multi-institutional studies in clinical settings, institution-related factors that may affect sleep quality should be identified and their influences determined.

## 8. Conclusions

The results of this study showed that sleep characteristics and sleep quality of most nurses in the transition to practice deteriorated. Such statistically significant changes in sleep characteristics were shown to be caused not only by a change in work structure, such as shift work, but also excess stress and work burden; consequently, the reduced quality of sleep had a statistically significant negative influence on the quality of life of new graduate nurses in the transition to practice.

## 9. Implications for Nursing Management

Institutions should improve existing work adaptation programs provided during new graduate nurses’ period of transition to practice by including information on the physical changes they may experience, with a particular focus on reducing the various negative consequences for the physical and psychological health of nurses of the changes in sleep characteristics and sleep quality accompanying their new role. Furthermore, as sleep characteristics or sleep quality of new graduate nurses are prone to changes caused by the work environment, efforts should be made to create a healthier work environment. As part of such efforts, more flexible scheduling should be promoted, and the maintenance of an appropriate workload level should be monitored.

## Figures and Tables

**Table 1:** Participants’ general characteristics (*N* = 88).

Characteristic	Category	*n* (%)
Age (years); mean = 23.25 ± 1.26	≤23	56 (64.4)
	24–25	26 (29.80)
	≥26	5 (5.8)
Sex	Female	71 (80.7)
	Male	17 (19.3)
Religion	Protestant	21 (23.9)
	Catholic	10 (11.4)
	Buddhist	4 (4.5)
	None	53 (60.2)
Hobbies	Yes	43 (49.4)
	Exercise ^†^	20 (11.9)
	Computer games ^†^	10 (41.1)
	TV, movie watching ^†^	8 (15.1)
	Travel ^†^	4 (10.8)
	Music or books ^†^	3 (21.1)
	No	44 (50.6)
Location of upbringing	Metropolitan area near Seoul	35 (41.2)
	Central area of Korea	13 (15.3)
	Southwestern area of Korea	6 (7.1)
	Southeastern area of Korea	28 (32.9)
	Other	3 (3.5)
Location of university	Metropolitan area near Seoul	21 (25.9)
	Northeastern area of Korea	6 (7.4)
	Central area of Korea	22 (27.2)
	Southwestern area of Korea	5 (6.2)
	Southeastern area of Korea	27 (33.3)
Work unit	Medical unit	39 (44.4)
	Surgical unit	37 (42.0)
	Emergence room	12 (13.6)
Grade point average	>4.0	19 (21.6)
	3.5–4.0	58 (65.9)
	<3.5	11 (12.5)

^†^ Double summation.

**Table 2:** Subjective health perception, sleep characteristics, and quality of sleep according to measurement time (graduate-transition) (*N* = 88).

Characteristic (Measurement Range)	Time	M ± SD (Reported Range)	95% Confidence Interval	*t* (*p*-Value)
Subjective health perception (1–5)	Before shift work	1.96 ± 0.55 (0.00–3.00)	−0.48, −0.17	−4.21 (*p* < 0.001)
	After shift work †	2.29 ± 0.62 (0.00–3.00)		
PSQI (Overall) (0–21)	Before shift work	6.33 ± 2.58 (1.00–15.00)	−1.82, −0.45	−3.32 (*p* = 0.001)
	After shift work †	7.47 ± 3.34 (2.00–17.00)		
Subjective sleep quality (0–3)	Before shift work	1.23 ± 0.60 (0.00–3.00)	−0.31, −0.13	−4.89 (*p* < 0.001)
	After shift work †	1.66 ± 0.70 (0.00–3.00)		
Sleep latency (min)	Before shift work	47.44 ± 37.87 (5.00–210.00)	−11.15, 4.72	−0.81 (*p* = 0.422)
	After shift work †	50.52 ± 40.13 (0.00–180.00)		
Sleep duration (h)	Before shift work	6.39 ± 1.76 (1.00–12.50)	−0.12, 0.80	1.47 (*p* = 0.145)
	After shift work †	6.05 ± 1.68 (2.00–10.00)		
Habitual sleep efficiency (0–100%)	Before shift work	88.43 ± 9.54 (50.00–98.97)	−1.13, 3.03	0.849 (*p* = 0.398)
	After shift work †	87.52 ± 10.22 (50.00–100.00)		
Sleep disturbance (0–27)	Before shift work	4.96 ± 3.30 (0.00–20.00)	−3.14,−1.08	−4.09 (*p* < 0.001)
	After shift work †	7.07 ± 4.44 (1.00–27.00)		
Use of sleep medication (0–3)	Before shift work	0.01 ± 0.11 (0.00–1.00)	−0.16, 0.02	−1.62 (*p* = 0.109)
	After shift work †	0.08 ± 0.38 (0.00–3.00)		
Daytime dysfunction (0–6)	Before shift work	1.12 ± 1.46 (0.00–5.00)	−0.82, −0.21	−3.32 (*p* = 0.001)
	After shift work †	1.63 ± 1.50 (0.00–6.00)		

M = mean; SD = standard deviation; PSQI = Pittsburgh Sleep Quality Index; † Four months after starting work as a new nurse.

**Table 3:** Quality of life according to measurement time (before and after shift work) (*N* = 88).

Characteristic (Measurement Range)	Time	M ± SD (Reported Range)	95% Confidence Interval	*t* (*p*-Value)
Quality of life (1–5)	Before shift work	3.75 ± 0.43	0.46, 0.68	10.49 (*p* < 0.001)
	After shift work †	3.18 ± 0.53		

† Four months after starting work as a new nurse.

**Table 4:** Influencing factors on the participants’ quality of life changes (*N* = 88).

Variables		Parameter Estimate	SE	*t* (*p*-Value)	95% Confidence Interval
Constant		5.47	1.02	5.38 <0.001	3.45, 7.48
Subjective health perception		−0.18	0.07	−2.39 0.018	−0.32, −0.03
Pittsburgh Sleep Quality Index	Subjective quality of sleep	−0.24	0.07	−3.43 0.001	−0.39, −0.10
	Sleep latency	−0.00	0.00	−1.33 0.188	−0.01, 0.00
	Sleep duration	0.04	0.03	1.13 0.260	−0.03, 0.10
	Habitual sleep efficiency	−0.01	0.01	−1.11 0.269	−0.04, 0.01
	Sleep disturbance	−0.00	0.01	−0.92 0.361	−0.03, 0.01
	Use of sleep medication	0.05	0.14	0.35 0.727	−0.23, 0.33
	Daytime dysfunction	−0.07	0.03	−2.42 0.017	−0.13, −0.01

SE = Standard Error.

## Data Availability

The datasets used and/or analyzed during the current study are available from the corresponding author on request.
